# Supplementary material for: The Influence of Body Composition, Lifestyle, and Dietary Components on Adiponectin and Resistin Levels and AR Index in Obese Individuals
Source: Int J Mol Sci. 2025 Jan 4;26(1):393. doi: 10.3390/ijms26010393 (PMC11720087; doi:10.3390/ijms26010393)
Supplement: Supplementary file 1 [file ijms-26-00393-s001.zip › File S1.pdf]

File S1. Original food consumption frequency questionnaire.

Diet and lifestyle survey:

Given name and surname:

1. Sex

a) female

b) male

2. Age …… years

3. Body weight …………. kg

4. Body height ……… cm

5. Place of residence

a) rural

b) urban

6. Assess your financial condition:

a) very poor

b) poor

c) good

d) very good

7. Diagnosed chronic diseases?

……………………………………………………………………………………………………......
....

8. Medication?

………………………………………………………………………………………………………
………………………………………………………………………………………

9. Diet supplements?

………………………………………………………………………………………………………
………………………………………………………………………………………

10. Are you drinking tea?

a) yes

b) no

11. If you answered yes in question 10, please describe what type of tea are you drinking and how frequent?

………………………………………………………………………………………………………
………………………………………………………………………………………

12. Are you drinking coffee?

a) yes

b) no

13. Are you use sugar for sweetening tea or coffee?

a) yes

b) no

14. Are you drinking water?

a) yes

b) no

15. How much fluid do you consume daily?

a) <0,5 liter

b) 0,5-1 liter

c) 1-2 liters

d) > 2 liters

16. Are you drinking alcohol?

a) yes

b) no

17. Are you salting foods and/or beverages?

a) yes

b) no

18. Are you using seasonings: Vegeta, Kucharek czy Maggi?

a) yes

b) no

19. How many meals do you eat per day?

a) 1-2

b) 3

c) 4-5

20. Do you eat between meals?

a) yes

b) no

21. What are the meal times?

……
………………………………………………………………………………………

22. How often do you eat these products? (mark X)

Products	Every day	4-5 times per week	2-3 times per week	once per week	1-2 times per month	I am not eating this product at all
White (e.g. wheat) bread						
Dark (e.g. rye, graham) bread						
Confectioneries e.g. donuts, buns, croissants						
Oat, barley and rye flakes						
Buckwheat, barley, millet						
White rice						
Brown rice						
Wheat noodles						
Whole grain pasta						
Potatoes						
Poultry (chicken, turkey)						
Red meat (pork, beef, lamb)						
Fishes						
Cold cuts, sausages, etc.						
Milk						
Sour natural milk products (buttermilk, kefir, yoghurt)						
Sour fruit milk products (buttermilk, kefir, yoghurt)						
White cheese e.g. cottage cheese						
Cheese						
Eggs						
Vegetables						
Fruits						
Cakes						
Chocolate bars, chocolates, etc.						
Fruit juices, nectars, fruit drinks						
Sweetened carbonated drinks e.g. Coke, Pepsi, Mirinda, Fanta, orangeade						
Alcohol						
Fast-food						

In the questions below you can choose more than one answer.

23. Where do you eat your meals?

a) at home

b) at work

c) in restaurants, canteens or fast foodzie spożywa Pan/Pani posiłki?

24. Which fats do you use for frying?

a) olive oil

b) sunflower oil

c) rapeseed oil

d) lard

e) butter

25. What do you use to spread your sandwiches?

a) butter

b) margarine

c) olive oil

d) fromage

26. What do you use for salads?

a) olive oil

b) rapeseed or sunflower oil

c) mayonnaise

d) yogurt

27. What are the most common heat treatment methods in your kitchen?

a) cooking

b) choking

c) frying

d) baking

28. Do you smoke cigarettes?

a) yes

b) no

29. Do you play sports??

a) yes

b) no

30. If you answered yes in question 29, please indicate what type of sport?

……
……
