# Supplementary material for: The Influence of Body Composition, Lifestyle, and Dietary Components on Adiponectin and Resistin Levels and AR Index in Obese Individuals
Source: Int J Mol Sci. 2025 Jan 4;26(1):393. doi: 10.3390/ijms26010393 (PMC11720087; doi:10.3390/ijms26010393)
Supplement: Supplementary file 1 [file ijms-26-00393-s001.zip › Supplementary Table S1.pdf]

Supplementary Table S1. Results of the statistical analysis between the frequency of consumption of nutrients and the level of adiponectin, resistin and AR index values.

Nutrients	Adiponectin [pg/ml]	Resistin [pg/ml]	AR index	Nutrients	Adiponectin [pg/ml]	Resistin [pg/ml]	AR index
Energy [kcal]	-0.07	0.02	-0.04	Vit. A [µg]	-0.02	0.04	-0.01
Protein [g]	-0.03	0.04	-0.03	Retinol [µg]	-0.06	0.07	-0.04
Fat [g]	0.12	-0.11	0.16	Beta carotene [µg]	-0.01	0.02	-0.01
Digestible carbohydrates [g]	-0.22 (p=0.042)	0.11	-0.22 (p=0.044)*	Vit. D [µg]	-0.07	0.04	-0.04
Fiber [g]	-0.04	0.08	-0.06	Vit. E [mg]	0.09	0.03	0.06
Vegetable protein [g]	-0.19	0.03	-0.19	Vit. K [µg]	-0.15	0.04	-0.13
Animal protein [g]	0.06	0.05	0.04	Vit. B1 [mg]	-0.07	0.01	-0.03
Sugars [g]	-0.07	-0.02	-0.01	Vit. B2 [mg]	-0.01	0.13	-0.03
Fructose [g]	-0.07	-0.10	-0.03	Vit. B3 [mg]	-0.06	0.03	-0.09
Galactose [g]	0.03	-0.04	0.07	Vit. B6 [mg]	-0.09	-0.02	-0.09
Glucose [g]	-0.07	-0.17	-0.01	Folates [µg]	-0.02	0.05	-0.02
Lactose [g]	-0.01	0.08	-0.03	Vit. B12 [µg]	0.02	-0.01	0.06
Maltose [g]	0.04	0.05	0.01	Vit. C [mg]	-0.16	-0.05	-0.15
Sucrose [g]	-0.05	0.02	-0.01	Isoleucine [mg]	-0.06	0.11	-0.08
Starch [g]	-0.27 (p=0.13)	0.12	-0.27 (p=0.012)*	Leucine [mg]	-0.03	0.14	-0.06
SFA [g]	0.20	-0.08	0.23 (p=0.036)*	Lysine [mg]	-0.02	0.12	-0.06
MUFA [g]	0.15	-0.18	0.22 (p=0.044)*	Methionine [mg]	-0.05	0.13	-0.08
n-3 fatty acids [g]	-0.03	0.11	-0.09	Cystine [mg]	-0.13	0.10	-0.13
n-6 fatty acids [g]	0.03	0.06	0.00	Phenylalanine [mg]	-0.03	0.11	-0.06
PUFA [g]	0.00	0.01	-0.01	Tyrosine [mg]	-0.02	0.13	-0.05
Cholesterol [mg]	-0.01	-0.05	0.03	Threonine [mg]	-0.04	0.11	-0.06
Trans-fatty acids [g]	-0.14	0.14	-0.18	Tryptophan [mg]	-0.05	0.10	-0.07
Sodium [mg]	-0.01	0.07	-0.01	Valine [mg]	-0.06	0.12	-0.08
Salt [g]	-0.01	0.07	-0.01	Arginine [mg]	-0.00	0.11	-0.01
Potassium [mg]	-0.04	0.04	-0.06	Histidine [mg]	-0.05	0.10	-0.08
Calcium [mg]	-0.09	0.13	-0.12	Alanine [mg]	-0.04	0.10	-0.07
Phosphorus [mg]	-0.01	0.12	-0.04	Kw. aspartic [mg]	-0.06	0.11	-0.09
Magnesium [mg]	-0.05	0.05	-0.08	Kw. glutamic [mg]	-0.04	0.15	-0.08
Iron [mg]	-0.04	0.06	-0.06	Glycine [mg]	-0.01	0.01	-0.01
Zinc [mg]	0.08	0.03	0.08	Proline [mg]	-0.06	0.11	-0.08
Copper [mg]	-0.13	0.10	-0,16	Serine [mg]	-0.04	0.15	-0.07
Manganese [mg]	0.14	0.03	0,11	GI	0.02	-0.11	0.01

Selenium [µg]	0.03	0.16	-0,04	GL	-0.21 (p=0.49)*	0.08	-0.20
Iodine [µg]	-0.07	0.10	-0,11	PRAL	0,03	-0.12	0.01

* red colour indicates significant values (p < 0.05)
